# Exploration of a new hepatitis a surveillance system in Beijing, China: based on molecular epidemiology

**DOI:** 10.1186/s12879-021-06872-4

**Published:** 2022-01-04

**Authors:** Huai Wang, Weixin Chen, Wenting Zhou, Feng Qiu, Wenjiao Yin, Jingyuan Cao, Pei Gao, Qianli Yuan, Min Lv, Shuang Bai, Jiang Wu

**Affiliations:** 1 Institute for Immunization and Prevention, Beijing Center for Disease Prevention and Control, Beijing Research Center for Preventive Medicine, No.16, He Ping Li Middle Street, Dongcheng District, 100013 Beijing, China; 2 NHC Key Laboratory of Medical Virology and Viral Diseases, National Institute for Viral Disease Control and Prevention, Chinese Center for Disease Control and Prevention, 102206 Beijing, China

**Keywords:** Hepatitis A virus, Molecular epidemiology, Surveillance, Transmission route

## Abstract

**Background:**

The incidence of hepatitis A virus (HAV) infection is low in Beijing, China, but the risk of outbreaks still exists. It is difficult to identify possible sources of infection among sporadic cases based on a routine surveillance system. Therefore, a more effective surveillance system needs to be established.

**Methods:**

The epidemiological data of hepatitis A were obtained from a routine surveillance system. Patients with HAV confirmed at the local hospitals were asked to complete a questionnaire that included additional case information and possible sources of infection. Serum and fecal specimens were also collected for testing HAV RNA by polymerase chain reaction. In addition, the 321-nucleotide segment of the VP1/2A junction region was sequenced to determine the HAV genotype.

**Results:**

In 2019, 110 HAV cases were reported in Beijing, with an incidence rate of 0.51/100,000. 61(55.5%) of these patients were male. The greatest proportion of these patients were aged from 30 to 60 years. The rate was lower in suburban and rural areas compared to urban areas. Contaminated food consumption, particularly seafood consumption, was the primary potential source of infection. Among the 16 specimens of confirmed HAV cases that could be sequenced, 93.8% were HAV IA, and 6.3% were HAV IB. In addition, the samples collected from all HAV sequences in this investigation showed 89.4–100% nucleotide homology. Two groups (each with three sporadic cases) showed 100% nucleotide homology. The three sporadic cases in one group had the same possible source of infection: contaminated salad with raw vegetables and seafood. In the other group, the three sporadic cases did not have an epidemiological connection.

**Conclusions:**

In a low HAV prevalent area, such as in Beijing, incorporating molecular epidemiology into the routine surveillance system could help inform possible clusters of outbreaks and provide support for earlier control of HAV transmission. Nevertheless, increased sampling from detected cases and improved specimen quality are needed to implement such a system.

**Supplementary Information:**

The online version contains supplementary material available at 10.1186/s12879-021-06872-4.

## Background

The prevalence of hepatitis A varies among different countries and is mainly associated with economic development, hygiene, environmental factors, and public education such as HAV transmission route and other HAV knowledge [1]. With the improvement of health conditions and the increase of HAV vaccination during childhood, the incidence of hepatitis A virus (HAV) infections has been dramatically reduced. Therefore, the incidence of HAV in Beijing, consistent with other developed countries, has been low in recent years. The reported incidence rate of HAV declined dramatically from 59.41/100,000 to 1990 to 0.80/100,000 in 2017 [2]. As a result, there has been no hepatitis A outbreaks in Beijing since 2004. However, similar to some developed countries, a high proportion of adults in Beijing are susceptible to HAV due to a decline in antibodies, which may result in possible outbreaks of hepatitis A [2–4].

In China, the National Notifiable Disease Reporting System (NNDRS) is a hospital-based passive surveillance system for infectious disease that can provide patient information, such as their age, sex, location of residence, date of onset, among other details [5]. Before 2003, HAV cases were reported on a monthly basis to the district Centers for Disease Control and Prevention (CDC) via hardcopy (case and hardcopy-based NNDRS) and then from the prefectural and provincial CDC to national authorities [5]. In 2004, the National CDC revised the reporting system by establishing an online electronic version (case and computer-based NNDRS). HAV cases could be directly reported from hospitals to the National CDC [5]. In Beijing, all local hospitals are involved in the NNDRS and are required to register cases of HAV if they find laboratory-confirmed or clinically suspected cases. However, outbreaks can only be detected after a certain number of cases are reported, resulting in a lag in results. Additionally, it is challenging to determine links between sporadic cases due to the long incubation period of HAV and delay in reporting HAV cases, therefore making it difficult to establish an association between contaminated food and illness.

HAV is the only member of the genus Hepatovirus in the Picornaviridae family. The HAV genome is a positive-stranded RNA of approximately 7.5 kb with an open reading frame that encodes a large polyprotein containing approximately 2230 amino acids. Unlike most RNA viruses, HAV has a low mutation rate over time, and therefore shows a high degree of antigenic and genetic conservation. Comparisons between nucleotide sequences allow us to genetically correlate different variants. If we could supplement routine epidemiological surveillance of HAV with molecular epidemiological studies, it may be possible to identify the isolates involved in outbreaks and the background variants from sporadic cases based on nucleotide variability [6].

The application of molecular methods in HAV surveillance was previously reported by other countries [7–9]. However, few studies on molecular epidemiological surveillance have been performed in China [10]. To establish a new molecular epidemiological surveillance system, we incorporated molecular epidemiology into the routine surveillance system. We surveyed HAV gene sequences from patients in Beijing in 2019 and described the possible clustered outbreaks using these molecular techniques. The objective of this report was to describe the incidence of HAV and the molecular epidemiological characteristics of patients with HAV in 2019, including sociodemographic variables, possible sources of infection, and molecular characteristics. This report also aimed to discuss the feasibility of the new molecular epidemiological surveillance system.

## Methods

### Epidemiological data collection and survey of potential sources of infection

Epidemiological data were collected through NNDRS in 2019. The cases were defined by the National Criteria and Principles of Management for Viral Hepatitis A (GB17010-1997) and National Diagnostic Criteria for Viral Hepatitis A (WS 298-2008). The clinical cases were tested for anti-HAV IgM by a local hospital. If positive anti-HAV IgM results were obtained, the hospital then reclassified the clinical case as a laboratory-confirmed case.

Patient information, including the name, age, sex, ID number, location of residence, date of onset, clinical symptoms, and hospital laboratory test results, were reported by the hospital through the NNDRS. Patients who tested positive for anti-HAV IgM and were diagnosed with hepatitis A by a local hospital were asked to complete a paper questionnaire. The questionnaire included additional case information, clinical symptoms, and more than one possible source of infection 15 to 45 days before the onset of symptoms. The clinical symptoms included fever, general malaise, loss of appetite, vomiting, nausea, aversion to greasy food, abdominal pain, pruritus, deep dark urine such as the color of tea, and jaundice. The possible sources of infection included vegetable and fruit consumption (raw or cooked, location), seafood consumption (uncooked or cooked, location), water (boiled or not), and details of travel. The questionnaire was collected by the staff of the district CDC.

### Sample collection

Two milliliters of serum and 5–10 g of feces were collected from patients who tested positive for anti-HAV IgM and were diagnosed with hepatitis A by a local hospital. All the samples were sent to the Beijing CDC and stored at − 20 ºC until RNA was extracted for testing.

### RNA extraction and RT–PCR

RNA extraction was performed from 100 µl of serum or fecal suspension with PBS after the addition of 500 µl of the TRIzol reagent (Invitrogen, Carlsbad, CA, USA) according to the manufacturer’s instructions. The RNA was dissolved in 20 µl of RNase-free water. cDNA was prepared by adding 5 µl of the extracted RNA to 25 µl of the RT mix according to the manufacturer’s instructions as follows: 5 µl of 5 × RT buffer, 5 µl of 2.5 mM dNTP mix, 1.5 µl of 10 µM reverse primer 3381, 1 µl of RNase inhibitor (20 U/µl, Promega, Madison, WI, USA) and 1 µl of AMV reverse transcriptase (10 U/µl, Promega). The RT mix was incubated at 42 ºC for 1 h and inactivated at 95 ºC for 5 min.

The VP1/2A junction region was amplified by PCR according to previous research [11]. The primers 2870 (position 2870, 5’-GAC AGA TTC TAC ATT TGG ATT GGT-3’) and 3381 (position 3381, 5’-CCA TTT CAA GAG TCC ACA CAC T-3’) were used with the exTaq polymerase (Takara, Dalian, China) according to the manufacturer’s instructions. Five microliters of the first PCR product were used with the primers 2897 (position 2897, 5’-GGT TTC TAT TCA GAT TGC AAA TTA-3’) and 3288 (position 3288, 5’-AAC TTC ATT ATT TCA TGC TCC T-3’) for the second nest PCR. PCR was performed mainly as follows: amplification for 35 cycles, denaturing for 30 s at 94 ºC, annealing for 30 s at 50 ºC, and elongation for 35 s at 72 ºC, followed by a final extension at 72 ºC for 7 min [12]. Sequencing was completed by Sangon Biotech Company(Sango Biotech, Shanghai, China).

### Sequence analysis

The sequences were processed using MegaX software (Molecular Evolutionary Genetics Analysis across computing platforms, Kumar, Stecher, Li, Knyaz, and Tamura 2018) [13]. The nucleotide sequences of the VP1/2A junction region of all the HAV variants were aligned with reference variants from different genotypes deposited in GenBank. The reference variants are shown in the Additional file 1 (Additional file 1: Table S1). A neighbor-joining (NJ) tree was constructed using the Kimura two-parameter correction method. Bootstrap resampling (1,000 pseudoreplicas) was used to assay the reliability of the analysis [14]. The HAV genotype groups were defined by <15% nucleotide (nt) variation, and the same subgenotypes were allowed to exhibit 0–7.5% nt variation [15]. The sequence data were used to determine the genotypes and to identify the source of infection. If the cases with the same sequence pattern and 100% homology can be considered to have the same source of infection such as same contaminated water, food or travel-related infections.

### Statistical analysis

EpiData (Version 3.1, http://www.epidata.dk/) was used to establish the databases. An additional epidemiological questionnaire for each patient was entered separately by two individuals from the CDC in Beijing. A third individual from Beijing CDC independently performed data checking. The data were analyzed using the statistical analysis package SPSS (SPSS Inc., Chicago IL, USA, version 19.0). MapInfo 9.5 software was used to build the HAV incidence map of 16 districts in Beijing.

## Results

### Incidence rate

**Fig. 1:**
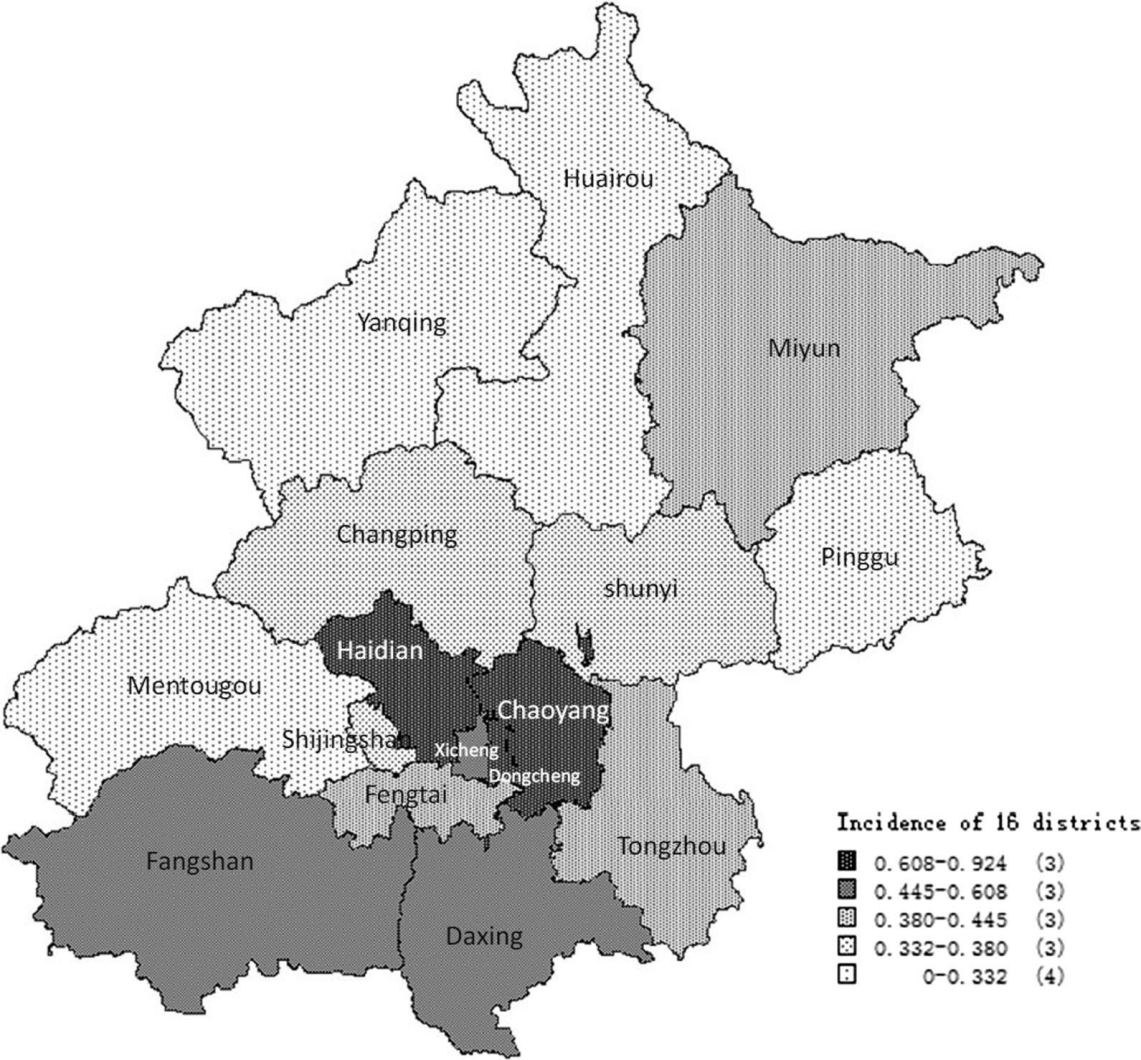
The incidence rate of HAV of 16 districts in Beijing in 2019

110 cases of HAV were reported in 2019, 61 (55. 5%) of the cases were male, and 72.7% were aged 30 to 60 years (Table 1). The incidence rate of HAV was 0.51/100,000 in 2019. The incidence map (Fig. 1, MapInfo 9.5 software) of the 16 districts in Beijing showed that the incidence rate of HAV ranged from 0/100,000 to 0.92/100,000. In addition, the incidence rate was lower in suburban and rural areas compared to urban areas.

**Table 1:** The number of reported HAV cases and incidence rate in 2019 in Beijing

	Incidence case (%)	Incidence rate (per 100,000)
Total	110	0.51
Gender		
Men	61 (55.5)	0.56
Women	49 (44.6)	0.46
Age		
0∼	0 (0.0)	0
15∼	1 (0.9)	0.20
20∼	2 (1.8)	0.17
25∼	4 (3.6)	0.15
30∼	13 11.8)	0.47
35∼	14 (12.7)	0.78
40∼	12 (10.9)	0.78
45∼	9 (8.2)	0.52
50∼	16 (14.6)	0.78
55∼	12 (10.9)	0.84
60∼	4 (3.6)	0.32
65∼	3 (2.7)	0.34
70∼	5 (4.6)	0.88
75∼	8 (7.3)	1.74
80∼	4 (3.6)	1.27
85∼	3 (2.7)	2.00

The data originated from NNDRS

### Potential sources of infection and clinical symptoms

110 patients with HAV confirmed at the local hospital were asked to complete the questionnaire. 90 patients completed the questionnaire(Table 2). The response rate is 81.8%. Among the 90 patients who completed the clinical symptoms and potential sources of infection questionnaire, 80 (88.9%) cases reported clinical symptoms. Seafood consumption (57.8%) and raw vegetable or fruit consumption (41.1%) were the primary potential sources of infection. Only 1 (1.1%) patient reported consuming nonboiled water, 8 (8.9%) patients reported traveling to other provinces in China, and 24 (26.7%) patients reported an unknown source of infection. The highest proportion of travel-related infections was found among middle-aged people.

**Table 2:** Clinical symptoms and a potential source of infection of HAV in Beijing

	N	Clinical symptoms (%)	Potential source of infection				
			Raw vegetable or fruit consumption (%)	Seafood consumption (%)	Nonboiled water (%)	Travelling (%)	Unknown (%)
Total	90	80 (88.9)	37 (41.1)	52 (57.8)	1 (1.1)	8 (8.9)	24 (26.7)
Gender							
Men	52	47 (90.4)	20 (38.5)	37 (71.2)	1 (1.9)	7 (13.5)	12 (23.1)
Women	38	33 (86.8)	17 (44.7)	15 (39.3)	0 (0.0)	1 (2.6)	12 (31.6)
Age							
20	3	3 (100.0)	3(100.0)	1 (33.3)	0 (0.0)	0 (0.0)	0 (0.0)
25∼	1	1 (100.0)	1(100.0)	1 (100.0)	0 (0.0)	0 (0.0)	0 (0.0)
30∼	9	8 (88.9)	4(44.4)	8 (88.9)	0 (0.0)	2 (22.2)	1 (11.1)
35∼	11	9(81.8)	3(27.3)	8 (72.7)	0 (0.0)	1 (9.1)	1 (9.1)
40∼	12	12(100.0)	5 (41.7)	9 (75.0)	1(8.3)	4 (33.3)	3 (25.0)
4∼	8	7 (87.5)	4 (50.0)	3 (37.5)	0 (0.0)	0 (0.0)	1 (12.5)
50∼	12	9 (75.0)	6 (50.0)	7 (58.3)	0 (0.0)	0 (0.0)	4 (33.3)
55∼	7	7 (100.0)	2 (28.6)	5 (71.4)	0 (0.0)	0 (0.0)	1 (14.3)
60∼	4	4 (100.0)	1 (25.0)	2 (50.0)	0 (0.0)	0 (0.0)	2 (50.0)
65∼	4	4 (100.0)	3 (75.0)	1 (25.0)	0 (0.0)	0 (0.0)	1 (25.0)
70∼	5	5 (100.0)	1 (20.0)	0 (0.0)	0 (0.0)	0 (0.0)	4 (80.0)
75∼	7	5 (71.4)	3 (42.9)	3 (42.86)	0 (0.00)	1 (14.3)	3 (42.9)
80∼	4	3 (75.0)	0 (0.0)	1 (25.0)	0 (0.0)	0 (0.0)	3 (75.0)
85∼	3	3 (100.0)	1 (33.3)	3 (100.0)	0 (0.0)	0 (0.0)	0 (0.0)

The data originated from clinical symptoms and potential sources of infection questionnaire

### Phylogenetic analysis of HAV sequences

A total of 68 in 110 (61.8%) patients with acute hepatitis A agreed to provide specimens. Among the 68 specimens, 16 (23.5%) could be sequenced. The HAV sequencing rate varied among districts (range from 0 to 75%). Among the 16 confirmed HAV case specimens that could be sequenced, 93.8% were HAV IA, and 6.3% were HAV IB. The genotypes found for the specimens from all three patients that reported traveling were IA.

**Fig. 2:**
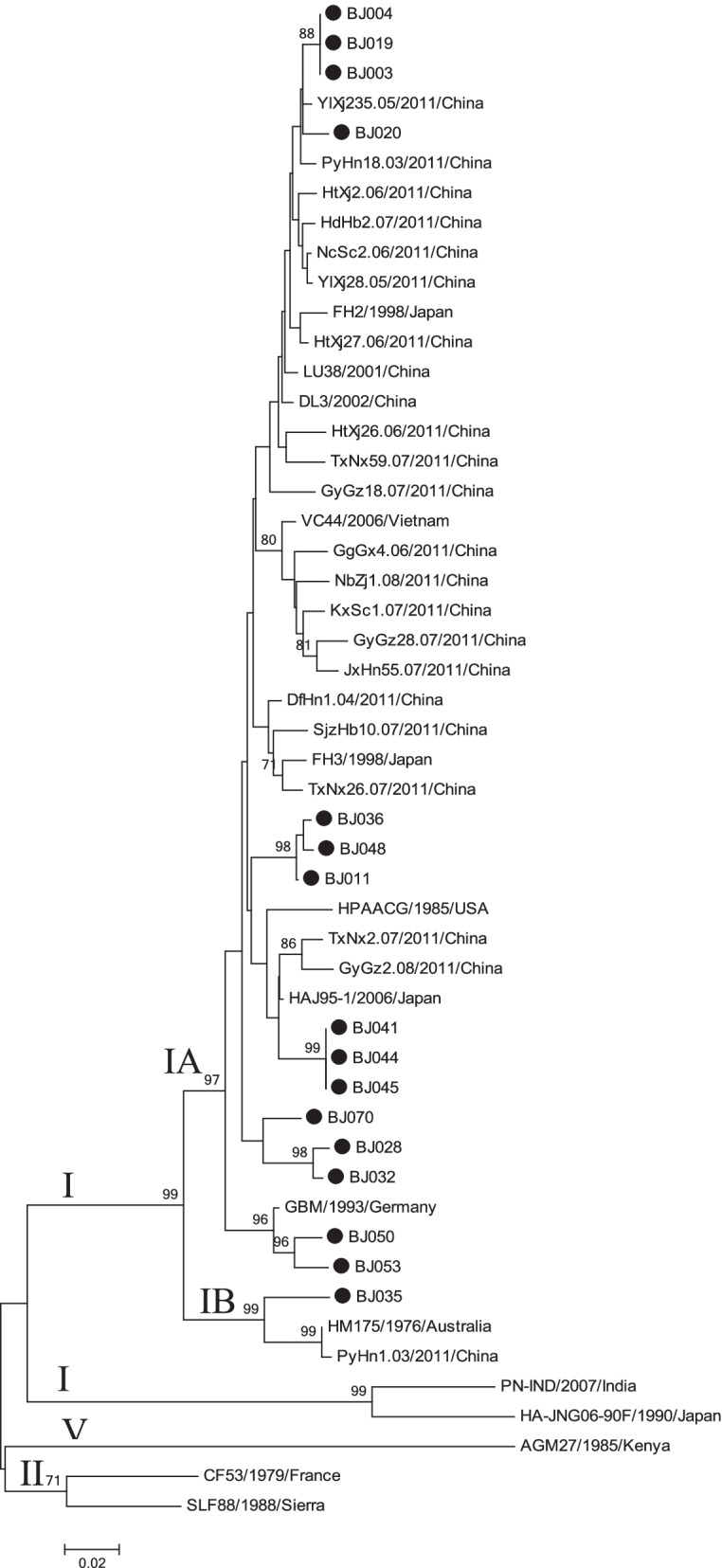
Phylogenetic analysis of HAV sequences obtained from Beijing in 2019. Nucleotide (nt) sequences from the VP1/2A junction region (321 bases) and 16 Beijing sequences from this study were analyzed by the neighbor-joining (NJ) method. The numbers at the nodes indicate bootstrap percentages over 1,000 replicates (only values >70% are shown). The bar length indicates genetic distance

The RT–PCR products of the VP1/2A junction region from the 16 Beijing HAV sequences were subjected to genotyping and phylogenetic analysis (Fig. 2). The results indicated that many HAV variants were circulating in Beijing. All sequences were classified into genotype I, and 15 of these sequences were further grouped into subgenotype IA. In addition, the results revealed 94.7–98.1% nucleotide sequence identity compared with the subgenotype IA reference variant GBM and 89.7–91.3% nucleotide sequence identity compared with the subgenotype IB reference variant HM175. Only one identical sequence was exclusively grouped into subgenotype IB, which showed 95.6% identity compared with the subgenotype IB reference variant HM175 and 90.7% nucleotide sequence identity compared with the subgenotype IA reference variant GBM. The 16 HAV sequences showed >15% nucleotide sequence differences in the VP1/2A junction region compared with HAV genotypes IIA, IIB, and V reference variants CF53, SLF88, and AGM27 from GenBank, respectively.

Pairwise comparisons of the nucleic acid sequences of these 16 HAV sequences revealed 89.4–100% homology in the VP1/2A junction region. Among the 16 sequences, one identical sequence was classified as subgenotype IB, and the remaining 15 sequences were further classified as subgenotype IA, showing 92.5–100% homology, and were clustered into five groups. The sequences BJ003, BJ004, and BJ019 showed 100% homology, and BJ041, BJ044, and BJ045 showed 100% homology. Subgenotype IA showed a 1.6–6.5% or 1.9–6.2% nucleotide sequence difference from the Chinese sequences LU38 or DL3 from GenBank, whereas a 4.5–6.9% or 1.9–6.2% nucleotide sequence difference was observed compared with BJ46.07 and BJ5.07, which were sequenced in 2011. A 2.9–7.6% nucleotide sequence difference was observed compared with the Japanese sequences HAJ95–1, FH2, or FH3, and a 3.2–6.6% nucleotide sequence difference was obtained compared with Vietnamese sequences VC44 from GenBank.

#### The epidemiological analysis of HAV cases revealed 100% homology

It is generally believed that cases with the same sequence pattern and 100% homology may generally be considered to have the same source of infection. The epidemiological history of one group with 100% homology (BJ003, BJ004, and BJ019) showed that the disease onset time was very close (December 2, 8, and 26 in 2019), but the patients did not live in proximity to one another. The patients also had different occupations, work units, and sexes. Had these patients no sequence data showing 100% homology, they would have been considered as sporadic cases. Further investigation revealed that these three patients all went to the same restaurant at a shopping center in Beijing around the same time, and the possible source of infection was contaminated salad with raw vegetables and seafood (the three patients all ate these foods).

In contrast, the epidemiological history of another group with 100% homology (BJ041, BJ044, and BJ045) showed that the disease onset time was not very close (June 23, July 22, and August 2). There were different sources of infection before the onset of disease, suggesting that detecting this epidemiological cluster would have been difficult if based solely on routinely collected surveillance data or sequence analysis.

## Discussion

Hepatitis A was a severe public health problem in China long ago, particularly in northern China [16, 17]. After introduction of the hepatitis A vaccine, the overall incidence rate of hepatitis A decreased, and less than 1 case per 100,000 individuals has been reported since 2008 [2]. The incidence rate of HAV was 0.51/100,000 in 2019, which is still low. All the cases were sporadic and mainly occurred in middle-aged individuals. The incidence rate was lower in suburban and rural areas compared to urban areas. In addition, 88.89% of patients reported clinical symptoms. Contaminated food consumption, especially seafood consumption, was the primary potential source of infection in Beijing. The highest proportion of travel-related infections was found among middle-aged individuals. Although the incidence rate is declining, the antibody-positive rate of people aged 15-20 years is less than 60% [2]. The spread of the disease and the regular accumulation of sufficiently susceptible people can cause periodic outbreaks. Therefore, it is important to strengthen surveillance. In Beijing, routine surveillance data could not identify any epidemiological clusters and all cases were considered as sporadic. It is difficult to determine the relationship and identify possible sources of infection among sporadic cases based on a routine surveillance system. Therefore, a more effective surveillance system needs to be established to identify possible outbreaks at early stages.

It is generally believed that cases with the same sequence pattern and 100% homology may generally be considered to have the same source of infection. If we can obtain the nucleic acid sequence of each case and identify 100% homologous sequences, we can link the sporadic cases and described the possible clustered outbreaks using these molecular techniques. The HAV subgenotypes IA and IB are both circulating in Beijing. Although different clusters or genetically close variants were observed, most of the sequences belonged to subgenotype IA. In this study, all the HAV nucleic acid sequences assayed at the VP1/2A junction (321 bases) showed 89.4 ~ 100% homology. In our study, two groups (each with three sporadic cases) showed 100% homology. Thus, a variety of genetically similar HAV variant genotypes or subgenotypes are prevalent in Beijing. Cao et al. genotyped acute HAV isolates from eight provinces in China from 2003 to 2008. All the isolates belonged to genotype I, 98.8% were clustered in subgenotype IA, and 1.2% were clustered in subgenotype IB [18].

This study incorporated molecular epidemiological methods into routine hepatitis A surveillance to improve our ability to describe possible clustered outbreaks and enhance our understanding of the molecular epidemiological characteristics of HAV in Beijing. This study is a pilot investigation of this new system and can suggest ways to further study individual outbreaks and improve these systems. This study included two groups with 100% homology. After an epidemiological survey, a group of patients was determined to have a history of eating at the same restaurant in a shopping center. The possible sources of infection were contaminated salad with raw vegetables and seafood which indicated the possibility of an outbreak and thus encouraged us to further investigate these contaminated foods. However, due to difficulties associated with food collection, no contaminated food was found in the restaurant to trace the source of infection. In most parts of the world, HAV testing in food is not part of routine analysis due to the disease’s long incubation period, and the related food is usually consumed or discarded before the infection is detected. Currently, Europe recommends using ISO 15216-1 to detect viruses in food [19]. China has also published the “Real-time RT–PCR method for the detection of norovirus and hepatitis A virus in exported food (SN/T 4784–2017) ” [20], but these methods require further evaluation. In some cases, the variability of the HAV nucleic acid sequence is limited to a certain extent, and relatively short sequencing fragments cannot accurately distinguish the genetic correlation of HAV isolates [6]. Homology might not always indicate a relationship between cases. This limitation must be weighed with the available epidemiological evidence. Therefore, we must combine epidemiology with molecular biology to make a sound judgment.

Although this study was able to address how molecular epidemiological surveillance can be used to enhance routine surveillance data in Beijing, there remain some limitations that deserve further discussion. First, a certain percentage of the respondents left several items in the questionnaire unanswered, which may lead to problems in identifying the possible sources of infection. We will strive to enhance the response rate in the future. Second, only a low proportion of the samples could be sequenced, which may be due to the length of time between disease onset and the sampling. Future studies should optimize the sampling process and shorten the sampling time. Moreover, a uniform anti-HAV IgM test will be needed in the future to avoid false-positive tests in different hospitals. Third, the new surveillance system has only been established for one year, and further studies are needed to develop a more comprehensive view of HAV infection in Beijing. Despite these problems, the new molecular epidemiological surveillance still could help inform possible clusters of outbreaks. Future studies should improve the response rate of the questionnaire and improve the proportion of the sequenced samples.

## Conclusions

In summary, the incidence rate of HAV in Beijing is low. Most of the patients presented clinical symptoms. Contaminated food consumption was the primary potential source of infection. The main HAV genotypes were IA and IB. The incorporation of molecular epidemiology into the routine surveillance system and the description of possible clustered outbreaks using these molecular techniques can be used to provide information for controlling HAV transmission. An effective surveillance system should include valid case background data, high-quality specimens, and sensitive detection methods. To establish a more effective surveillance system, we should strive to improve the data and specimen quality, and adopt a sensitive method.

## Supplementary Information

**Additional file 1: Table S1.** HAV reference variantsand GenBank ascension number

## Data Availability

The data used and/or analyzed for the current study are available from the corresponding author upon reasonable request.

## Abbreviations

NNDRS: National Notifiable Disease Reporting System
HAV: Hepatitis A virus
CDC: Centers for Disease Control and Prevention
