# Supplementary material for: Exploration of a new hepatitis a surveillance system in Beijing, China: based on molecular epidemiology
Source: BMC Infect Dis. 2022 Jan 4;22:22. doi: 10.1186/s12879-021-06872-4 (PMC8725380; doi:10.1186/s12879-021-06872-4)
Supplement: Supplementary file 1 — Additional file 1: Table S1. HAV reference variantsand GenBank ascension number [file 12879_2021_6872_MOESM1_ESM.docx]

**Additional file 1**

Table S1. HAV reference variants and GenBank ascension number

| Reference variants | GenBank number | Reference variants | GenBank number | Reference variants | GenBank number |
| --- | --- | --- | --- | --- | --- |
| YlXj235.05 | HQ907933 | LU38 | AF357222 | KxSc1.07 | HQ907938 |
| PyHn18.03 | HQ907925 | DL3 | AF512536 | GyGz28.07 | HQ907919 |
| HtXj2.06 | HQ907935 | HtXj26.06 | HQ907936 | JxHn55.07 | HQ907926 |
| HdHb2.07 | HQ907928 | TxNx59.07 | HQ907917 | DfHn1.04 | HQ907923 |
| NcSc2.06 | HQ907927 | GyGz18.07 | HQ907918 | SjzHb10.07 | HQ907931 |
| YlXj28.05 | HQ907932 | VC44 | AB253604 | FH3 | AB020569 |
| FH2 | AB020568 | GgGx4.06 | HQ907922 | TxNx26.07 | HQ907916 |
| HtXj27.06 | HQ907934 | NbZj1.08 | HQ907937 | HPAACG | K02990 |
| TxNx2.07 | HQ907915 | GyGz2.08 | HQ907920 | HAJ95-1 | AB258583 |
| GBM | X75215 | HM175 | M14707 | PyHn1.03 | HQ907924 |
| PN-IND | EU011791 | HA-JNG06-90F | AB258387 | AGM27 | D00924 |
| CF53 | AY644676 | SLF88 | AY644670 |  |  |
